# Relationship between physical exercise and perceived stress in university students: a cross-sectional study on the chain mediating role of social support and general self-efficacy

**DOI:** 10.3389/fpsyg.2026.1836913

**Published:** 2026-08-20

**Authors:** Hao Sun, Yihu Zhao

**Affiliations:** School of Sports, Shandong University of Finance and Economics, Jinan, Shandong, China

**Keywords:** chain mediation, general self-efficacy, perceived stress, physical exercise, social support

## Abstract

**Objective:**

This study aimed to explore the association between physical exercise and perceived stress among college students and its underlying psychological transmission pathways, and to examine the independent and chain mediating roles of social support and general self-efficacy in this relationship.

**Methods:**

A cross-sectional study design was adopted, and a questionnaire survey was conducted among 750 college students from 5 universities in a Chinese city. The SPSS macro PROCESS (Model 6) was applied to test the chain mediation model at the 95% confidence interval via 5,000 bootstrap resamplings.

**Results:**

Physical exercise was significantly and negatively associated with perceived stress (β = −0.367, 95% CI [−0.429, −0.304]). The total indirect effect of social support and general self-efficacy was −0.225 (95% CI [−0.270, −0.185]), accounting for 61.31% of the total effect. Specifically, three indirect pathways were identified: physical exercise → social support → perceived stress (indirect effect = −0.034, 9.26% of the total effect), physical exercise → general self-efficacy → perceived stress (indirect effect = −0.155, 42.23% of the total effect), and the chain mediation pathway of physical exercise → social support → general self-efficacy → perceived stress (indirect effect = −0.036, 9.81% of the total effect).

**Conclusion:**

College students‘ participation in physical exercise is significantly associated with lower levels of perceived stress, and social support and general self-efficacy play a key chain mediating role in this relationship. This study theoretically reveals the internal psychological mechanism of the association between physical exercise and perceived stress, and provides empirical reference for universities to implement mental health improvement interventions. This study empirically elucidates the possible mechanisms underlying the association between physical exercise and perceived stress, providing empirical reference for intervention practices aimed at improving college students' mental health in higher education settings.

## 1 Introduction

Mental health issues have become a major challenge in the global public health domain. In recent years, multiple cross-cultural epidemiological studies have indicated a significant upward trend in the prevalence of anxiety, depression, and high perceived stress among youth populations. College students are at a critical stage of physical and mental development and social role transition, facing multiple stressors such as academic development, career planning, and interpersonal relationships. Surveys show that over 90% of college students in China experience varying degrees of perceived stress (Chen et al., 2022). Long-term high levels of perceived stress are prone to induce negative emotions such as anxiety and depression, and even trigger psychological problems, which severely impair individuals‘ academic performance and long-term development. Alleviating student stress and promoting physical and mental health has become a widespread concern across society (Sheldon et al., 2021). Therefore, exploring low-cost, highly accessible non-pharmacological intervention strategies to reduce college students' stress and promote wellbeing has become a core topic for scholars in psychology, education, and public health worldwide.

Among numerous intervention approaches, physical exercise—as a health behavior with both physiological and psychological benefits—has gained widespread recognition in the international academic community. According to exercise psychology theory, physical exercise plays a positive role in regulating attentional orientation, improving cognitive function, and modulating emotional states. However, previous studies have predominantly focused on physiological mechanisms or framed exercise merely as a means of physical fitness, relatively overlooking the educational value of sports and the psychological and social transmission pathways behind it as a complex social behavior.

Social support and general self-efficacy are key factors shaping individuals‘ perceived stress. As a critical external protective resource, social support can buffer the negative impact of stressful events on mental health (Wang et al., 2020). As a core internal belief, general self-efficacy directly influences individuals' appraisal and coping styles toward stressful events (Dou et al., 2013). Although existing studies have separately examined the mediating roles of the two variables in the link between physical exercise and mental health outcomes, few have integrated both into a unified framework to investigate their sequential mediating effects in the relationship between physical exercise and perceived stress.

Accordingly, this study constructs a chain mediation model of “physical exercise → social support → general self-efficacy → perceived stress” among college students to empirically delineate the internal pathways through which physical exercise alleviates perceived stress.

## 2 Theoretical basis and hypotheses

### 2.1 The association between physical exercise and perceived stress

The core dependent variable of this study is perceived stress, defined as individuals' subjective appraisal of stressful events, challenges, and threats in their environment, along with the resulting psychological tension—a core indicator of mental health status. The core independent variable is physical exercise, referring to planned, organized, and regular physical activity aimed at enhancing physical fitness and promoting physical and mental health, serving as the key intervention variable in this study.

In the field of sports science, abundant research has confirmed the positive effect of physical exercise on perceived stress. Physiologically, physical exercise is associated with increased endorphin secretion, reduced cortisol levels, and lower perceived stress levels (Li et al., 2015). Psychologically, the experience of mastering skills and achieving goals during exercise helps individuals improve psychological adaptability and willpower, optimizes cognitive appraisal of stress-inducing events, and lowers perceived stress levels (Ma and Yan, 2010). In school sports research, studies have pointed out that regular physical exercise can effectively alleviate college students' negative emotions and stress related to academics and employment (Chen, 2023). Thus, Hypothesis 1 (H1) is proposed:

1. H1: Physical exercise is negatively correlated with perceived stress in college students.

### 2.2 The mediating effect of social support

Social support refers to the resources individuals obtain through social connections that mitigate psychological stress responses, relieve mental tension, and improve social adaptability (Li, 1998). If college students can subjectively perceive and actively utilize their social resources, they can reduce emotional disturbances such as high anxiety and depression caused by low interpersonal trust, poor peer relationships, and low self-esteem. Greater social support enables them to cope with difficulties better and improves their adversity resilience, thereby successfully alleviating perceived stress (Wang et al., 2024). Social support exerts a universal health-promoting effect; it maintains individuals‘ positive emotional experiences and physical wellbeing in daily life, rather than only functioning under obvious stress, thus benefiting mental health (Xue and Wang, 2012). College is a critical period for individuals to obtain social resources and establish social connections; if individuals fail to utilize their social support system or establish one, they are prone to various problems. Hence, social support is a vital environmental factor determining students' adaptability (Hu, 2013). Evidently, for college students, social support is a critical factor in relieving perceived stress, maintaining mental health, and influencing personal growth.

As a social behavior and mode of interpersonal interaction, physical exercise is positively correlated with individuals' social support (Ge et al., 2023). From the perspective of social interaction theory, physical exercise, as a social behavior, can facilitate interpersonal communication and elevate the level of received social support. Studies have found that during physical exercise, students continuously learn to communicate and cooperate with others, which subtly cultivates their interpersonal skills and cooperative awareness, enhancing their social support levels (Lu et al., 2024). Thus, Hypothesis 2 (H2) is proposed:

1. H2: Social support plays a mediating role in the relationship between physical exercise and college students' perceived stress.

### 2.3 The mediating effect of general self-efficacy

General self-efficacy refers to individuals‘ global belief in their ability to cope with diverse environmental challenges and accomplish various tasks, an extension and application of Bandura's self-efficacy theory in general contexts (Guo and Jiang, 2003). General self-efficacy theory posits that individuals' behavioral outcomes are determined by their self-efficacy; the stronger their general self-efficacy, the more they can persist in their behavioral expectations while dealing with various challenges and risks (Liu, 2020).

Physical exercise has a positive impact on general self-efficacy. Empirical studies have shown that active participation in physical exercise significantly improves college students‘ general self-efficacy (Zhang, 2015). Concurrently, general self-efficacy can effectively buffer college students' stress; once students form strong self-confidence, even when facing significant pressure, they will not experience severe perceived stress (Guo et al., 2023). Based on this, this study suggests that physical exercise first enhances individuals‘ general self-efficacy, which in turn alleviates college students' perceived stress through its protective role. Thus, Hypothesis 3 (H3) is proposed:

1. H3: General self-efficacy plays a mediating role in the relationship between physical exercise and perceived stress.

### 2.4 The chain mediating effect of social support and general self-efficacy

Beyond their independent mediating roles, social support and general self-efficacy may function sequentially in the relationship between physical exercise and perceived stress. According to the Conservation of Resources (COR) theory, individuals tend to acquire and protect the resources they value, and external social resources, such as social support, can effectively promote the accumulation of internal psychological resources like general self-efficacy (Hobfoll, 2001). According to Bandura's social cognitive theory, self-efficacy beliefs are shaped by four primary sources: mastery experiences, vicarious experiences, verbal persuasion, and emotional arousal. In the context of physical exercise, social support—particularly encouraging feedback from peers, coaches, and teammates—serves as a powerful form of verbal persuasion, enhancing individuals‘ confidence in their capabilities. Additionally, observing similar peers succeed in physical activities provides vicarious experiences that further strengthen self-efficacy beliefs. This theory clarifies why social support functions not merely as a direct stress buffer but also as a catalyst for building individuals' internal psychological resources (Bandura, 2012).

In the context of physical exercise, research provides ample support for this chain pathway. Physical exercise, as a typical social interaction platform, can significantly expand college students' social networks and elevate their perceived social support levels (Guo et al., 2024). When college students receive peer encouragement and team acceptance during exercise, this high-quality external social support not only directly buffers stress but also translates into positive psychological suggestions, significantly boosting their confidence in coping with daily academic and life challenges—namely, enhancing general self-efficacy (Wang et al., 2025).

Furthermore, individuals with abundant social support and high general self-efficacy tend to adopt more proactive self-management and coping strategies when facing multiple stressors (Zhang et al., 2024), rather than falling into emotional depletion, ultimately significantly reducing their perceived stress experiences (Wilski et al., 2024). Therefore, physical exercise can not only independently associate with perceived stress through social support and general self-efficacy, respectively but also exert its effect through the chain mediation mechanism of social support and general self-efficacy. Thus, Hypothesis 4 (H4) is proposed:

1. H4: Social support and general self-efficacy play a chain mediating role in the relationship between physical exercise and college students' perceived stress.

**Figure 1:**
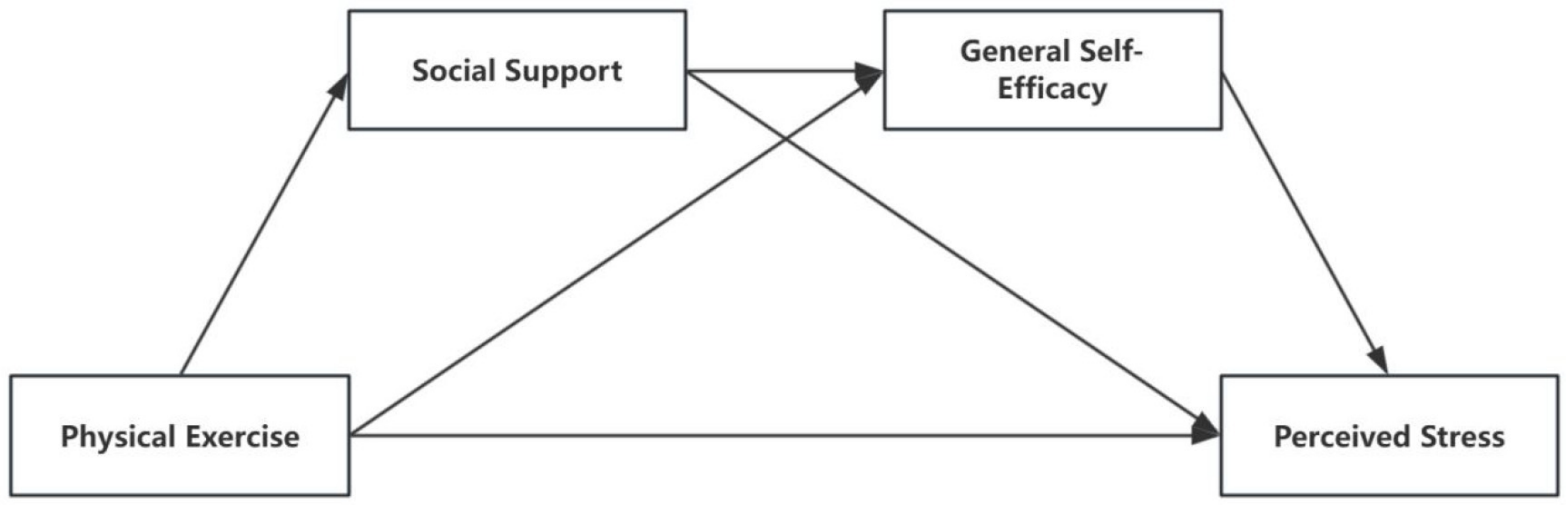
Hypothetical model diagram.

Therefore, this study constructs a chain mediation model (Figure 1) to systematically examine the sequential pathways through which physical exercise may be associated with perceived stress.

## 3 Subjects and methods

### 3.1 Participants

This study focuses on the association between physical exercise and college students' perceived stress levels, as well as the mediating roles of social support and self-efficacy. A stratified cluster random sampling method was adopted. Specifically, five universities in a certain city were first stratified according to their institutional characteristics (comprehensive, normal, and engineering universities). From each stratum, one university was randomly selected, and within each selected university, 3–4 classes were randomly chosen from different grades and majors. The five participating universities included one comprehensive university, two normal/liberal arts universities, one engineering university, and one university of finance and economics. Before distributing the questionnaires, students were asked to read the instructions to understand the purpose and significance of the study; beginning the survey constituted informed consent, guaranteeing anonymity and not involving minors. This survey was distributed via an online platform, yielding a total of 750 questionnaires. After excluding questionnaires based on three criteria—(1) incorrectly answering trap questions and reverse-scored questions, (2) selecting identical options for all items, and (3) showing patterned responses, 704 valid questionnaires were finally obtained, yielding an effective rate of 93.87% (348 males accounting for 49.43%, 356 females accounting for 50.57%; 345 Arts students accounting for 49.01%, 261 Science students accounting for 37.07%, and 98 Art and PE students accounting for 13.92%). Using G^*^Power 3.1 for sample size estimation, setting the effect size *f* ^2^ = 0.15, significance level α = 0.05, and statistical power 1-β = 0.95, the minimum sample size required for multiple regression analysis was 138. The valid sample size of 704 in this study is far higher than the minimum sample size requirement, fully meeting the testing power requirements for the statistical analysis in this study.

### 3.2 Research instruments

#### 3.2.1 Physical exercise rating scale

The Physical Exercise Rating Scale revised by Liang (1994) was adopted, which contains three items designed to assess physical activity levels, covering exercise intensity, duration, and frequency. Each item is scored on a 5-point Likert scale. Specifically, the scoring range for exercise intensity and frequency is 1 to 5 points, while the scoring range for exercise duration is 0 to 4 points. The total physical activity score is calculated by the product of exercise intensity, duration, and frequency; a higher total score indicates a higher degree of participation in physical activity. In this study, its Cronbach's α was 0.745.

#### 3.2.2 Social support scale

The college students Social Support Scale developed by Ye and Dai (2008) was used to measure social support. The scale contains 17 items across three dimensions: objective support, subjective support, and support utilization. Items are rated on a 5-point Likert scale (1 = completely disagree, 5 = completely agree); higher total scores indicate higher social support. A sample item: “I often receive care and support from friends and classmates.” In this study, overall Cronbach's α = 0.899; dimension Cronbach's α values were 0.817 (objective support), 0.829 (support utilization), and 0.825 (subjective support), indicating excellent reliability.

#### 3.2.3 General self-efficacy scale

The General Self-Efficacy Scale compiled by Schwarzer and revised by Wang et al. (2001) was adopted to measure college students' general self-efficacy levels. The scale consists of 10 items, using a 5-point Likert scoring method, where 1 represents “completely incorrect” and 5 represents “completely correct”; a higher total score on the scale represents a higher level of general self-efficacy for the individual. A representative item is “With my ingenuity, I can definitely cope with unexpected situations”. In this study, the Cronbach's α coefficient of the scale was 0.930.

#### 3.2.4 Perceived stress scale

The Chinese Perceived Stress Scale (CPSS) revised by Yang and Huang (2003) was used to measure college students' perceived stress levels. This scale is the most widely used standardized tool in the field of perceived stress research in China, comprising 14 items, using a 5-point Likert scoring method, where 1 represents “never” and 5 represents “always”; a higher total score on the scale represents a higher level of perceived stress by the individual. A representative item is “I feel that I cannot handle all the important things in my life”. In this study, the Cronbach's α coefficient of the scale was 0.916.

### 3.3 Statistical methods

Excel was used for data entry, cleaning, and invalid questionnaire screening. SPSS 27.0 was used for statistical analysis. Model 6 of the PROCESS 4.1 macro (Hayes, 2013) was applied to test the significance of mediating effects via bias-corrected bootstrap with 5,000 resamples and a 95% confidence interval. An effect is considered statistically significant if its 95% bootstrap confidence interval does not contain zero.

## 4 Results and analysis

### 4.1 Common method bias test

Due to the influence of measurement environment, questionnaire instructions, and context, questionnaire data may exhibit common method bias (Zhou and Long, 2004). Harman's single-factor test was conducted on the measurement data. Unrotated principal component analysis using SPSS 27.0 on physical exercise, perceived stress, social support, and general self-efficacy revealed 7 factors with eigenvalues greater than 1. The variance explained by the first factor was 30.646%, which is below the 40% critical threshold. On this basis, the common method factor (CMF) method was further applied; results showed that after adding the common method factor, the average change in item factor loadings was less than 0.1, further confirming that no severe common method bias existed in this study.

### 4.2 Descriptive statistics and correlation analysis

The means, standard deviations, and Pearson rank correlation coefficient matrices of the core variables are shown in Table 1. The results showed: physical exercise was significantly and positively correlated with social support and general self-efficacy (*r* = 0.222, 0.490, *P* < 0.01), and significantly and negatively correlated with perceived stress (*r* = −0.398, *P* < 0.01); social support was significantly and positively correlated with general self-efficacy (*r* = 0.508, *P* < 0.01), and significantly and negatively correlated with perceived stress (*r* = −0.416, *P* < 0.01); general self-efficacy was significantly and negatively correlated with perceived stress (*r* = −0.583, *P* < 0.01). Pairwise correlations among all variables reached a significant level, fulfilling the statistical prerequisites for mediation effect analysis and providing preliminary support for the hypotheses of this study.

**Table 1:** Means, standard deviations, and Pearson correlations among key variables (*N* = 704).

Variables	M	SD	1	2	3	4
1. Physical exercise	2.892	0.703	–			
2. Social support	3.536	0.503	0.222^**^	–		
3. General self-efficacy	3.667	0.735	0.490^**^	0.508^**^	–	
4. Perceived stress	2.493	0.648	−0.398^**^	−0.416^**^	−0.583^**^	–

^*^P < 0.05, ^**^P < 0.01, ^***^P < 0.001.

### 4.3 Direct and indirect effect test of physical exercise on perceived stress

This study took physical exercise as the independent variable, perceived stress as the dependent variable, and social support and general self-efficacy as mediating variables to construct a chain mediation model. The regression analysis results are shown in Table 2. The regression analysis results showed:

**Table 2:** Regression analysis results of physical exercise, social support and general self-efficacy on perceived stress.

Variables	Perceived stress		Social support		General self-efficacy		Perceived stress	
	β	*t*	β	*t*	β	*t*	β	*t*
Physical exercise	−0.367	−11.478^***^	0.159	6.037^***^	0.416	13.323^***^	−0.141	−4.468^***^
Social support					0.613	14.083^***^	−0.216	−4.837^***^
General self-efficacy							−0.372	−10.895^***^
*R* ^2^	0.398		0.222		0.639		0.614	
Δ*R*^2^	0.158		0.049		0.048		0.377	
*F*	131.737^***^		36.447^***^		241.534^***^		141.104^***^	
Cohen's *f*^2^	0.611		0.285		1.770		1.591	

^***^P < 0.001.

The negative predictive association of physical exercise on perceived stress was significant (β = −0.141, *P* < 0.001), and H1 was established.

The positive predictive association of physical exercise on social support was significant (β = 0.159, *P* < 0.001), and the negative predictive association of social support on perceived stress was significant (β = −0.216, *P* < 0.001), and H2 was established.

The positive predictive association of physical exercise on general self-efficacy was significant (β = 0.416, *P* < 0.001), and the negative predictive association of general self-efficacy on perceived stress was significant (β = −0.372, *P* < 0.001), and H3 was established.

The predictive association of social support on general self-efficacy was significant (β = 0.613, *P* < 0.001). Combining the above conclusions, it indicates that there is a significant chain mediating effect of social support and general self-efficacy between physical exercise and perceived stress, and H4 was established.

Additionally, the Cohen's *f* ^2^ effect sizes for the four regression models were 0.661, 0.285, 1.770, and 1.591, respectively, all exceeding the large-effect threshold of 0.35 indicating that the models had substantial practical significance beyond statistical significance.

Next, the mediation effect test was conducted. The Bootstrap test with 5,000 repeated samplings was used to respectively test the mediating effects of social support and General self-efficacy between physical exercise and perceived stress as well as the confidence intervals, taking the upper and lower limits of the 95% confidence interval not containing 0 as the criterion for a significant effect, as shown in Table 3. The results showed:

**Table 3:** Path analysis of the chain mediation model.

Path	Effect value	Boot SE	Boot 95% CI Lower Limit	Boot 95% CI Upper Limit	Proportion of total effect
Total effect	−0.367	0.032	−0.429	−0.304	100%
Direct effect	−0.141	0.032	−0.203	−0.079	38.42%
Total indirect effect	−0.225	0.021	−0.270	−0.185	61.31%
Ind1: Physical exercise -> social support -> perceived stress	−0.034	0.009	−0.054	−0.018	9.26%
Ind2: Physical exercise ->general self-efficacy -> perceived stress	−0.155	0.020	−0.195	−0.117	42.23%
Ind3: Physical exercise ->social support ->general self-efficacy -> perceived stress	−0.036	0.007	−0.052	−0.023	9.81%

The total effect value of physical exercise on perceived stress was −0.367 (LLCI=-0.429, ULCI=-0.304), indicating that the total effect of physical exercise on perceived stress and the mediating effects of the two variables were significant.

The direct effect value of physical exercise on perceived stress was −0.141 (LLCI = −0.203, ULCI = −0.079), indicating that physical exercise had a significant direct association with perceived stress, and the effect value accounted for 38.42% of the total effect.

Social support and General self-efficacy each played a mediating effect between physical exercise and perceived stress (with three paths in total), and the total indirect effect value was −0.225 (LLCI = −0.270, ULCI = −0.185), accounting for 61.31% of the total effect. Among them, the first mediation effect path: physical exercise → social support → perceived stress (Path 1), the indirect effect value was −0.034 (LLCI = −0.054, ULCI = −0.018), accounting for 9.26% of the total effect; The second path: physical exercise → General self-efficacy → perceived stress (Path 2), the indirect effect value was −0.155 (LLCI = −0.195, ULCI = −0.117), accounting for 42.23% of the total effect; The third chain mediation effect path: physical exercise → social support → General self-efficacy → perceived stress (Path 3), the indirect effect value was −0.036 (LLCI = −0.052, ULCI = −0.023), accounting for 9.81% of the total effect.

**Figure 2:**
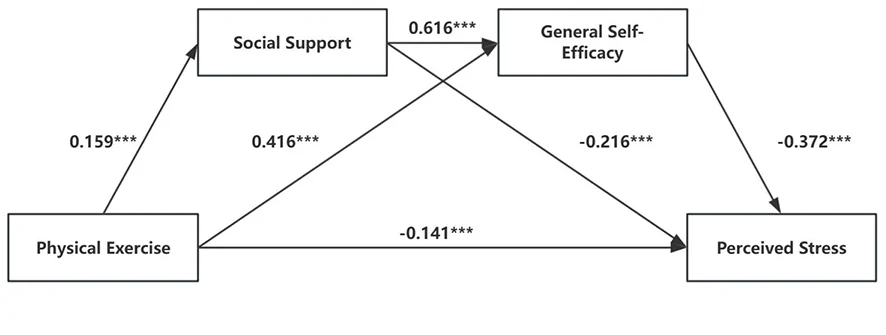
Final chained mediation model of physical exercise on college students' perceived stress. * *P* < 0.05, ** *P* < 0.01, *** *P* < 0.001.

Based on the above research results, the chain mediation model is shown in Figure 2.

## 5 Discussion

Based on cross-sectional data, this study constructed and validated a chain mediation model of social support and general self-efficacy in the association between physical exercise and perceived stress among college students. The findings not only confirm the direct protective association of physical exercise with perceived stress but also disentangle its underlying social and cognitive transmission pathways.

### 5.1 The relationship between physical exercise and perceived stress

Physical exercise exhibited a significant negative association with college students' perceived stress, and this direct effect accounted for a substantial proportion. This result echoes previous findings, further verifying the intrinsic link between regular exercise and lower stress levels, and providing empirical evidence for the research consensus. According to the Cognitive-Phenomenological-Transactional (CPT) theory model, stress is not a singular external stimulus or an isolated emotional response (Bramwell et al., 2023), but rather a dynamic evolutionary process of continuous interaction between the individual and the environment. Its formation mechanism revolves around a two-stage cognitive appraisal of external situations, specifically encompassing primary appraisal and secondary appraisal, which are sequential and mutually influential (Peluso and Guerra de Andrade, 2005).

In the primary appraisal stage, individuals evaluate the nature of external stressful events to determine whether they pose potential threats, cause actual loss, or present developmental challenges. Aligning with existing literature, regular physical activity is believed to modulate individuals' cognitive processing biases, helping to reduce excessive attention to negative information and catastrophic interpretations. This behavioral habit may prompt individuals to view external stressors from a calmer perspective, thereby being associated with a reduction in the subjective threat perception of academic, interpersonal, and developmental stress events, demonstrating a buffering potential at the cognitive source (Wu et al., 2022). Upon entering the secondary appraisal stage, individuals judge whether they can effectively cope with the identified stressful situations based on their available physical and psychological resources; this appraisal profoundly impacts the ultimate level of perceived stress. During this stage, the dual physical and mental benefits accumulated through long-term exercise may exert a positive adaptive effect, further alleviating perceived stress levels (Liu et al., 2025). Therefore, for college students in early adulthood facing multiple real-world pressures—such as deep academic study, career planning, and social adaptation—physical exercise is not merely a routine activity for physical health but may also serve as a crucial non-pharmacological protective factor, playing an active adaptive role in daily stress buffering and mental health maintenance.

### 5.2 The mediating role of social support

Social support played an independent mediating role between physical exercise and perceived stress. Consistent with prior studies, this indicates that physical exercise may be linked to lower perceived stress by enhancing interpersonal interactions and elevating individuals‘ perceived social support. From a social interaction perspective, physical exercise possesses the dual attributes of bodily activity and social engagement (Eime et al., 2013). Campus-based participation forms, such as PE classes and sports clubs, provide low-pressure interpersonal settings for students; individuals engaging in physical activities might more easily gain peer acceptance and positive interpersonal feedback in such scenarios, thereby improving their social support levels (Herbert et al., 2020). Simultaneously, according to the classic “Stress-Buffering Hypothesis,” social support—a widely validated protective factor for mental health—is significantly and negatively correlated with perceived stress. Sufficient external support resources can effectively buffer the negative impacts of stressful events (e.g., academic or employment pressure) on individuals' psychological states (Dong et al., 2024). The sequential linkage of these two aspects constitutes the theoretical and empirical foundation for this mediation pathway.

### 5.3 The mediating role of general self-efficacy

General self-efficacy exerted the largest independent mediating effect between physical exercise and perceived stress, with its effect size far exceeding that of both the independent and chain mediating effects of social support. This suggests that one of the core mechanisms linking physical exercise to lower perceived stress levels lies in its reconstruction of an individual's internal belief system. According to Bandura's social cognitive theory (Bandura, 1997), self-efficacy beliefs are primarily shaped by four sources: mastery experiences, vicarious experiences, verbal persuasion, and emotional arousal. Physical exercise may provide individuals with rich and repetitive mastery experiences. As individuals continuously challenge their physical limits, break through personal barriers, and master new motor skills in sports, they undergo a cognitive transition from “I cannot” to “I can.” These mastery experiences acquired through physical practice may possess strong domain generality, implying that the confidence and efficacy built in the sports domain can potentially transfer to other life domains, such as academics, socializing, and career development (White et al., 2017). When students internalize the self-efficacy honed during physical exercise into a generalized belief, they may be more inclined to adopt proactive problem-solving strategies rather than passive avoidance when confronting academic setbacks or employment anxiety (Gloria Steinhardt, 2016), thereby reducing their subjective perception of stress to a certain extent.

### 5.4 The chain mediating role of social support and general self-efficacy

This study verified the sequential chain pathway “physical exercise → social support → general self-efficacy → perceived stress”, indicating that social support and general self-efficacy may exert a sequential chain mediating effect in this association. Specifically, social support positively predicted general self-efficacy, which provides empirical backing for the Conservation of Resources theory. This theory posits that external resources (like social support) can effectively facilitate the accumulation of internal resources (like self-efficacy).

In the context of campus sports participation, this chain mechanism may unfold as follows: college students may garner social support—such as peer encouragement, coach validation, and teammate acceptance—through participating in physical exercise. These positive feedbacks constitute verbal persuasion and vicarious experiences within Bandura's framework, potentially assisting individuals in gradually dispelling self-doubt and establishing intrinsic confidence. Thus, they experience a psychological transition from “relying on external resources” to “generating internal confidence,” ultimately facilitating a reduction in stress experiences. This chain conversion mechanism from “external resources” to “internal resources” demonstrates that the association between physical exercise and perceived stress likely involves both social interaction and cognitive reconstruction dimensions, rather than relying on a singular physiological or psychological pathway.

## 6 Research conclusions, practical implications, and limitations & prospects

### 6.1 Research conclusions

Through empirical analysis, this study reached the following main conclusions:

All pairwise correlations among the four variables—physical exercise, social support, general self-efficacy, and perceived stress—reached statistically significant levels among college students.

Physical exercise possesses a significant negative direct association with college students' perceived stress, potentially serving as an effective means to alleviate it.

Social support and general self-efficacy each play a significant independent mediating role between physical exercise and perceived stress.

Social support and general self-efficacy exert a significant chain mediating effect between physical exercise and perceived stress. Physical exercise can indirectly alleviate college students' perceived stress through this chain pathway.

### 6.2 Practical implications

The chain mediation model in this study reveals the intrinsic mechanisms by which physical exercise mitigates college students' perceived stress. The following practical implications are provided as references for college mental health education and physical education teaching.

First, carry out physical education reforms centered on enhancing general self-efficacy. This study found that the mediating effect of general self-efficacy accounts for 42.23% of the total effect, making it the strongest among the three pathways. This implies that physical exercise is associated with lower perceived stress largely because it helps students establish the internal belief of “I can do it.” Therefore, college PE curricula should not merely target motor skill proficiency or fitness test scores, but should emphasize allowing students to gain continuous “success experiences” during exercise. Specifically, PE teachers can adopt layered, progressive instructional designs, setting personalized milestone goals for students with different athletic backgrounds to ensure every student can make progress. Simultaneously, course assessments should shift from single competitive outcomes to process-oriented evaluations focusing on effort and improvement margin, aiding students in transferring sports-built confidence into their academic and daily lives.

Second, expand students' social support networks by leveraging campus sports activities. This study found that the independent mediating effect of social support accounts for 9.26% of the total effect, and its chain mediating effect accounts for 9.81%, totaling approximately 19%. The social nature of campus sports activities provides a natural interaction platform for students. Universities can organize class leagues, sports clubs, and interest groups to help students build peer support relationships during exercise. When students encounter stressful events in life, these social connections established through physical exercise can serve as crucial support resources.

Third, integrate physical exercise as a routine intervention method into mental health education. The direct effect in this study accounts for 38.42% of the total effect, indicating that physical exercise itself has a direct association with lower perceived stress levels. Universities can utilize physical exercise as an auxiliary intervention for mental health education by lowering the participation barrier and cultivating regular exercise habits through forms like campus sports APP check-ins, sports culture festivals, and morning run plans. For students identified with high stress levels during psychological screenings, guidance into appropriate physical activities could be attempted as a supplementary intervention pathway alongside psychological counseling.

### 6.3 Limitations and future prospects

This study has several limitations to be addressed in future research. First, the cross-sectional design only demonstrates associations, not causal relationships. Longitudinal tracking or randomized controlled intervention trials with multi-month exercise programs are needed to establish causality and examine dynamic changes in mediation pathways. Second, all data were self-reported. Despite controls for common method bias, social desirability and recall bias may remain. Future studies should incorporate multi-source evaluations (peer, teacher ratings) and objective physiological markers (heart rate variability, salivary cortisol) to cross-validate stress levels. Third, demographic variables (gender, grade, major) were not included as covariates. Although preliminary analyses showed no stress differences across these groups, future studies should control for them to rule out confounding effects. Fourth, this study did not distinguish between group and individual exercise, or examine differences in social support mechanisms across exercise modes. Future research can explore whether individual exercise also confers social support benefits in the digital age. Finally, the sample was limited to five universities in one city, restricting generalizability. Future studies should expand sampling to diverse regions and institution types, and examine potential moderators such as region, school type, and major to refine the theoretical model.

## Data Availability

The raw data supporting the conclusions of this article will be made available by the authors, without undue reservation.
